# The natural triterpene maslinic acid induces apoptosis in HT29 colon cancer cells by a JNK-p53-dependent mechanism

**DOI:** 10.1186/1471-2407-11-154

**Published:** 2011-04-27

**Authors:** Fernando J Reyes-Zurita, Gisela Pachón-Peña, Daneida Lizárraga, Eva E Rufino-Palomares, Marta Cascante, José A Lupiáñez

**Affiliations:** 1 Department of Biochemistry and Molecular Biology I, University of Granada, 18071 Granada, Spain; 2 Department of Biochemistry and Molecular Biology, University of Barcelona, 08028 Barcelona, Spain; 3 Department of Health Risk Analysis and Toxicology, Maastrich University, Maastrich, The Netherlands

**Keywords:** colon-cancer cells, JNK, maslinic acid, mitochondrial apoptotic pathway, p53-mediated apoptosis, triterpenes

## Abstract

**Background:**

Maslinic acid, a pentacyclic triterpene found in the protective wax-like coating of the leaves and fruit of *Olea europaea *L., is a promising agent for the prevention of colon cancer. We have shown elsewhere that maslinic acid inhibits cell proliferation to a significant extent and activates mitochondrial apoptosis in colon cancer cells. In our latest work we have investigated further this compound's apoptotic molecular mechanism.

**Methods:**

We used HT29 adenocarcinoma cells. Changes genotoxicity were analyzed by single-cell gel electrophoresis (comet assay). The cell cycle was determined by flow cytometry. Finally, changes in protein expression were examined by western blotting. Student's t-test was used for statistical comparison.

**Results:**

HT29 cells treated with maslinic acid showed significant increases in genotoxicity and cell-cycle arrest during the G0/G1 phase after 72 hours' treatment and an apoptotic sub-G0/G1 peak after 96 hours. Nevertheless, the molecular mechanism for this cytotoxic effect of maslinic acid has never been properly explored. We show here that the anti-tumoral activity of maslinic acid might proceed via p53-mediated apoptosis by acting upon the main signaling components that lead to an increase in p53 activity and the induction of the rest of the factors that participate in the apoptotic pathway. We found that in HT29 cells maslinic acid activated the expression of c-Jun NH2-terminal kinase (JNK), thus inducing p53. Treatment of tumor cells with maslinic acid also resulted in an increase in the expression of Bid and Bax, repression of Bcl-2, release of cytochrome-c and an increase in the expression of caspases -9, -3, and -7. Moreover, maslinic acid produced belated caspase-8 activity, thus amplifying the initial mitochondrial apoptotic signaling.

**Conclusion:**

All these results suggest that maslinic acid induces apoptosis in human HT29 colon-cancer cells through the JNK-Bid-mediated mitochondrial apoptotic pathway via the activation of p53. Thus we propose a plausible sequential molecular mechanism for the expression of the different proteins responsible for the intrinsic mitochondrial apoptotic pathway. Further studies with other cell lines will be needed to confirm the general nature of these findings.

## Background

The activation of apoptotic pathways is a key mechanism by which anticancer drugs kill tumor cells [1,2]. Anticancer drugs normally induce apoptosis signaling via two major pathways: the mitochondrial or intrinsic pathway, and the death-receptor or extrinsic pathway. The intrinsic pathway involves the release of pro-apoptotic factors such as cytochrome-c from the mitochondria, which activate the apoptotic mechanism by interacting with Apaf-1 and stimulating the initiator caspase-9, which in turn induces proteolytically the activity of executor caspase-3, one of the principle proteases participating in the execution phase of apoptosis.

In the extrinsic pathway, activation of the death receptor stimulates the activation of the initiator caspase-8, which then triggers downstream events either by directly activating caspase-3 or by cleaving the Bid factor, which in turn initiates the mitochondrial pathway. Nevertheless, Bid has also been seen to be activated by JNK [3,4]. Bid-active targets the mitochondria to modulate other Bcl-2-like factors such as Bax [5]. It is not clear, however, whether Bid is the only target of pro-apoptotic JNK signaling. Death caused by over-expressed MKK7-JNK proteins has in fact been shown to require the Bax-like factor of the Bcl-2 group [6]. Furthermore, JNK may act directly upon the Bcl-2 protein family, thus inducing the mitochondrial pathway. JNK phosphorylates members of the Bcl-2 family of proteins, such as Bcl-2, and inactivates their apoptotic function [7]. Moreover, the expression of constitutively active JNK (using the fusion protein MKK7-JNK1) efficiently induces apoptosis in wild-type cells but not in cells lacking the pro-apoptotic Bcl-2 family member, such as Bax [6]. The activation of JNK has been described as being necessary for the induction of apoptosis in different cell types [6,8].

Apart from this, JNK also phosphorylates and regulates the activity of transcription factors such as p53 [9,10]. It has also been reported that in response to UV, c-Jun (the principal target of JNK) inhibits p53-mediated cell-cycle arrest, thereby promoting p53-mediated apoptosis [11]. It is well known that c-Jun functions as a direct repressor of p53 gene transcription [12]. In addition, it activates at least two proteins in the intrinsic pathway, including Bax and the p53 apoptosis-inducing factor. In human leukemia cells, different anticancer drugs increase p53 phosphorylation and the induction of JNK pathways [13].

Several mechanisms for the induction of apoptosis by p53 have been identified, involving both the transcriptional and/or non-transcriptional regulation of its downstream effectors. For example, p53 induces apoptosis by transcriptional up-regulation of pro-apoptotic genes such as Bax, and by transcriptional repression of anti-apoptotic Bcl-2 [14]. It has also been found that p53 translocates to mitochondria before the release of cytochrome-c and the activation of pro-caspase-3 [15]. It is also reported to induce apoptosis via physical interaction with the anti-apoptotic proteins Bcl-2 and Bcl-xL at their DNA-binding domains, thus leading to the sequestration of these proteins from their interaction with pro-apoptotic partners such as Bax/Bak proteins, which as a result may form oligomers and permeabilize the outer mitochondrial membrane, thereby causing the release of mitochondrial cytochrome-c into the cytosol [16].

The natural triterpenoid maslinic acid (MA) is the main component (80%) of the protective wax-like coating of olives. It has anti-oxidant, anti-inflammatory and anti-tumoral properties [17,18]. We have recently described the effects of these properties [19] but until now the precise mechanism for its activity had not been determined. The participation of JNK in triterpene-induced apoptosis has been reported previously. For example, CDDO activates JNK in U-937 leukemia cells [20] and the same authors obtained similar results with CDDO imidazolide ester and CDDO methyl ester. CDDO-Me induces apoptosis via JNK-mediated DR up-regulation in human lung-cancer cells [21]. The stimulation of JNK, activation of caspase-8, loss of mitochondrial transmembrane potential, release of cytochrome-c and cleavage of caspase-3 have all been described in triterpene-induced apoptosis [22].

We describe here the genotoxicity induced by MA as observed by comet assays, in which chromatin integrity is quantified at the level of single cells. We also evaluated apoptosis induction and cell-cycle arrest. Finally, we determined the probable molecular mechanism via which MA induces its cytotoxic effects in HT29 cells. In this way have seen how maslinic acid activates p53 and thus helps to trigger apoptosis. We determined how, in HT29, MA activates MAP kinases, including JNKs and thus p53, by which means they increase their functional activity. We further found that MA's capacity to trigger apoptosis depends upon JNK and p53 via the induction of Bax, the inhibition of Bcl-2, the release of cytochrome-c, the activation of caspase-9 and finally that of caspases -3, -7 and -8. Further studies with different colon-cancer cell lines will be needed to validate the general nature of the proposed mechanism.

## Methods

### Materials

Dulbecco's modified Eagle's medium (DMEM), bisbenzimide (Hoechst 33258), low-melting-point agarose (LMP), high-melting-point agarose (HMP), phosphate-buffered saline (PBS) and propidium iodide (PI) were from Sigma (St. Louis, MO, USA). Foetal calf serum (FCS) and penicillin/streptomycin were from Gibco-BRL (Germany). 4',6-diamidino-2-phenylindole (DAPI) was from Molecular Probes (Invitrogene, Eugene, USA). Primary antibodies, anti-caspase-3, -9 and anti-JNK were from Cell Signaling Technology (USA). Anti-caspase-7, -8 and anti-Bid were from Biosciences, Belgium. Anti-p53 was from Calbiochem (Germany). Anti-Bax, anti-Bcl-2, anti-cytochrome-c and secondary antibodies were from Santa Cruz Biotechnology (California, USA). All other reagents were of analytical grade.

### Drugs and cells

Maslinic acid is a triterpene derivate from pressed olives. Its molecular weight is 472.7 g/mol. The extract used was a powder comprising 98% maslinic acid and 2% oleanolic acid, which is stable when stored at 4°C. It was dissolved before use at 10 mg/mL in 25% DMSO and 75% PBS. A stock solution was frozen and stored at -20°C. Prior to experiments this solution was diluted in cell-culture medium. All experiments were conducted at IC50 = 61 ± 1 µM and IC80 = 76 ± 1µM, the values of MA concentrations required for 50% and 80% growth inhibition after 72 hours' treatment [18]. Human colorectal adenocarcinoma cell line HT29 (ECACC no. 91072701) was cultured in DMEN medium supplemented with 2 mM glutamine, 10% heat-inactivated FCS, 10,000 units/mL penicillin and 10 mg/mL streptomycin. Subconfluent monolayers of cells were used in all experiments.

### Single-cell gel electrophoresis (comet assay)

We adapted the single-cell gel-electrophoresis assay to assess DNA integrity. The technique, commonly known as the comet assay, is a sensitive way of measuring DNA single-strand breaks at the level of single cells. Cells were suspended in 160 µL of 0.5% LMP in PBS at 37°C and pipetted onto a frosted-glass microscope slide precoated with 1% HMP in PBS. The agarose was allowed to set in a thin layer on the microscope slide by protecting it with a coverslip at 4°C for 10 min. The slides were then immersed in a lysis solution (1% Triton X-100, 2.5 M NaCl, 0.1 M EDTA, and 10 mM Tris 1% lauryl sulphate, pH 10.0) at 4°C for 1 h to remove any proteinaceous materials while leaving "nucleoids", i.e. supercoiled DNA that is morphologically similar to that found in intact cell nuclei. After lysis the slides were placed in a wide horizontal electrophoresis tank containing 0.3 M NaOH and 1 mM EDTA at 4°C for 20 min to promote the unwinding of DNA at damaged sites before electrophoresis. During alkaline electrophoresis at 25 v for 30 min, DNA was attracted to the anode; segments of unwound DNA were free to migrate, extending from the nucleoid head to form the tail of a comet-like image. After being washed with buffer (0.4 M Tris-HCl, pH 7.5) the slides were stained for 15 min with DAPI to detect DNA single-strand breaks. Assessments were made using a fluorescence microscope equipped with an excitation filter of 450-490 nm. Tail moment values were quantified by computational scoring of 100 randomly selected comets per gel [23].

### Flow cytometric analysis

Flow cytometry is used as a diagnostic tool to measure DNA ploidy as well as to measure alterations in cell cycle profiles characteristic of DNA fragmentation (necrosis) compared to patterned DNA cleavage (apoptosis). The information obtained includes the visualization of cell subpopulations with differing DNA contents. For each nucleus subpopulation identified, the parameters of population size, fractions of nuclei in each phase of the cell cycle and computation of DNA ratios can be discerned. DNA hypoploid changes are characteristic of apoptosis. Cells seeded at a density of 175 × 103 HT29 cells/well were plated in 6-well plates with 2 mL of medium. After 24 h the cells were treated with different doses of MA for 72 or 96 h. The cells were resuspended by trypsinization and washed in PBS. The cells (6 × 105) were fixed in ice-cold ethanol (70%) for 30 min. The total DNA content was stained with 1 mg/mL PI. The number of cells at each stage of the cell cycle was estimated by fluorescence-associated cell sorting (FACS) and monitored by flow cytometry. The cell cycle was analyzed using Multicycle software. The data were analyzed to determine the percentage of cells at each phase of the cell cycle (G0/G1, S and G2/M) or in aneuploid peak.

### Cytosolic extracts for cytochrome-c analysis

HT29 cells (1.2 × 106) were cultured in 100-mm plates and treated for 48 and 72 h with MA at concentrations of IC50 and IC80. After treatment the cells were washed with ice-cold PBS and resuspended in ice-cold lysis buffer (20 mM HEPES, pH 7.5, 10 mM KCl, 1.5 mM MgCl2, 1 mM EDTA, 1 mM EGTA, 250 mM sucrose, 1 mM dithiothreitol, 0.1 mM phenyl methylsulfonyl fluoride, 1 μg/mL pepstatin A, 2 μg/mL leupeptin and 10 μg/mL aprotinine). After incubation on ice for 30 min the cells were homogenized with 15 strokes of a pre-chilled pestle B homogenizer and the homogenates were centrifuged at 25,000 g for 30 min at 4°C. The supernatants were further centrifuged at 25,000 g for 30 min at 4°C and stored at -80°C for cytochrome-c analysis.

### Western blotting

The HT29 cells (1.2·106) were treated with MA at IC50 and IC80 for 12, 24, 48 and 72 h, after which they were washed twice with PBS and resuspended in lysis buffer (20 mM Tris/acetate, pH 7.5, 270 mM sucrose, 1 mM EDTA, 1 mM EGTA, 1% Triton X-100, 1 mM ortovanadate, 1 mM sodium glycerophosphate, 5 mM sodium fluoride, 1 mM sodium pyrophosphate, 5 mM β-mercaptoethanol, 1 mM bezamidine, 35 μg/mL PMSF, 5 μg/mL leupeptine). The samples were homogenized ultrasonically and incubated on ice for 20 min before being centrifuged at 12,000 g for 15 min. The supernatants were assayed for protein concentration.

For western-blot analyses, a 25-50 μg sample of total proteins was separated on 15% SDS-polyacrylamide gel and transferred to a polyvinylidene difluoride membrane. The membranes were blocked by incubation in TBS buffer containing 0.1% Tween-20 and 5% dried milk for 1 h at room temperature and washed with TBS buffer containing 0.1% Tween-20. They were then blotted overnight at 4°C with primary antibodies [rabbit polyclonal anti-caspase-3 and -9 (1/1000 dilution) and mouse monoclonal anti-JNK (1/500 dilution), rabbit anti-caspase-7 and anti-Bid (1/3000 dilution), rabbit anti-Bax and mouse monoclonal anti-Bcl-2 (1/500 dilution), mouse monoclonal anti-p53 (1/200 dilution)]. To determine caspase-8, membranes were blotted for 1 h at 25°C with a rabbit polyclonal primary antibody anti-caspase-8 (1/3000). The blots were washed 3 times with TBS-0.1% Tween and developed with peroxidase-linked secondary antibodies (1/3000). For cytochrome-c determination, membranes were blotted for 1 h at 25°C with a mouse monoclonal primary antibody anti-cytochrome-c (1/3000). All blots were developed by ECL Western Blotting Detection Kit Reagent and detected using a LAS-3000 imaging system.

### Statistics

Data are given as the means ± SEM. For each assay Student's *t-test *was used for statistical comparison with the untreated control cells. A limit of p ≤ 0.05 was accepted for significant differences.

## Results

### Single-cell gel electrophoresis (comet assay)

**Figure 1:**
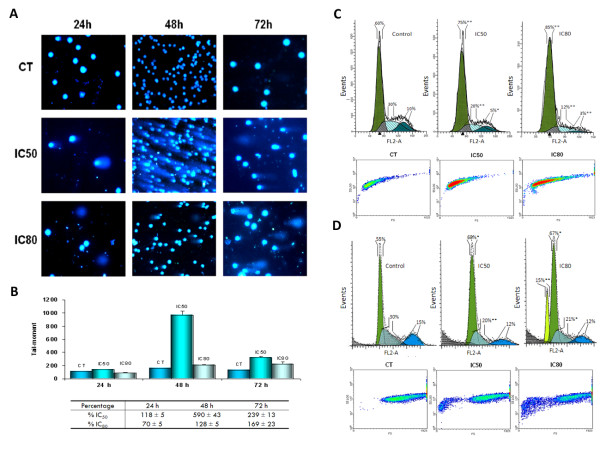
**(Panel A) Comet assay images of HT29 cells treated with maslinic acid after 24, 48 and 72 h at concentrations of IC_50 _and IC_80_**. An increase in the tail moment can be seen after 48 hours' treatment at IC_50_. (Panel B) Tail-moment values observed in HT29 cells treated with maslinic acid at concentrations of IC_50 _and IC_80 _after 24, 48 and 72 h. Percentages compared to untreated control cells are also shown. The values represent means ± SD of four separate experiments. (Panel C) Histograms of cell cycle of HT29 cells after 72 hours' treatment with maslinic acid. Diagram of forward scatter (FS) versus side scatter (SS) of HT29 cells. It can be seen that the central population corresponding to cells in the G0/G1 cell cycle phase was increased. (Panel D) Histograms of cell cycle of HT29 cells after 96 hours' treatment with maslinic acid. A hypodiploid peak can be seen in the sub-G0/G1 region. Diagram of FS versus SS of HT29 cells. Sub-G0/G1 populations are seen to appear at both IC_50 _and IC_80_. Values represent means of three separate experiments. Significances are represented as (*) P < 0.05, and (**) P < 0.01.

We determined DNA integrity in single cells by the comet assay (Figure 1A). The results indicated that MA interfered with DNA integrity in HT29 cells. To verify the sensitivity and reliability of the comet assay in detecting DNA damage we conducted tests that showed that treatment of cells by peroxide or MMS produced a significant increase in the tail moment (data not shown). Three incubation times (24, 48 and 72 h) and two concentrations of MA (IC50 and IC80) were tested to determine clastogenic effects (Figure 1B). HT29 cells treated with MA showed no significant increase in clastogenic effects after 24 h (only 18% at IC50). After 48 h at IC50, however, these values increased almost 6-fold. At IC80 a lesser increase in the tail moment was observed at all treatment times, with no effect after 24 h, 28% after 48 h and 69% after 72 h, probably due to the cytotoxic effect induced by maslinic acid under these conditions.

### Flow cytometric analysis

In the light of the inhibition of cell growth caused by MA reported by [18] we investigated its effects on cell-cycle distribution. The proliferation and proportions of cells in different phases of the cell cycle were analyzed at 72 and 96 h by the incorporation of PI. DNA histogram analysis revealed that MA induced a concentration-dependent increase in the number of cells within the G0/G1 phase with a maximum at IC80 (21.4 ± 6.0%). This increase was accompanied by a decrease in the percentage of proliferating cells (17.7 ± 5.4% in phase S plus 3.3 ± 1.4% in phase G2/M) (Figure 1C). At IC50 the increase in cells in the G0/G1 phase was 12.5 ± 5.1%, and the decrease in phase S was 9.4 ± 4.4% and 3.7 ± 1.1% in phase G2/M. This concentration-dependent increase in the G0/G1 phase can also be seen in the increase in the central population of FS/SS.

Flow cytometry after PI staining allows cell-cycle analysis, and any cells undergoing apoptosis can be detected as a subdiploid peak. The results revealed that after 96 h of IC80 maslinic treatment, the population of HT29 cells detected in the sub G0/G1 peak increased substantially (Figure 1D). At IC80 the hypodiploid region included 15% of total events. In this case the appearance of a population of cell fragments in the sub-G0/G1 phase was evident both at IC50 and IC80, a result that could clearly be seen in the FS/SS analysis.

### Maslinic acid induces the expression of JNK and p53

Therapeutic agents trigger apoptosis via the activation of stress-related signaling pathways including p53 and JNK-mediated ones and so we examined the possibility that MA might activate the JNK pathway. We found that MA did indeed induce an apoptotic mitochondrial activation via JNK, including cleavage of Bid and the mitochondrial translocation of Bax and Bcl-2 inhibition. JNK activation was determined by western blotting at 12, 24, 48 and 72 h. The results showed that MA highly induces the activation of JNK in HT29 cells.

**Figure 2:**
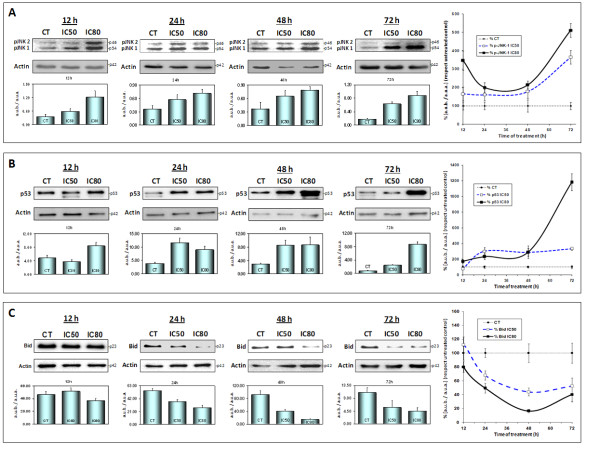
**Western blottings of the levels of JNK (panel A), p53 (panel B) and Bid (panel C) proteins**. HT29 cells were treated with maslinic acid at concentrations of IC_50 _and IC_80 _for 12, 24, 48 and 72 h. The levels of protein expression are expressed as arbitrary intensity units of each band compared to arbitrary intensity units of actin. The variations in relative percentages of the expression of proteins for each time and concentration are also shown. The values represent means ± SD of at least three separate experiments.

As shown in Figure 2A, JNK induction increased concomitantly with dosage and incubation time, beginning after 12 hours' treatment at both concentrations of IC50 and IC80. A relative maximum level of JNK expression was reached after 12 h at IC80 (2.5-fold). The maximum levels of JNK expression were reached after 72 h at IC80 and IC50 (4-fold at IC80 and 2.6-fold at IC50). It appears that the JNK pathway is induced 12 h after the cells are exposed to MA, followed by the transcription of p53 between 12 and 24 h and then the activation of caspase-8 between 48 and 72 h, followed finally by the appearance of apoptotic bodies at 96 h. Thus the triggering of the JNK pathway is an early event in the apoptotic chain and its activation plays a central role in the up-regulation of apoptosis exerted by maslinic acid.

Activation of the p53 signal pathway in HT29 cells after treatment with MA was tested by western blotting (Figure 2B) at 12, 24, 48 and 72 h. A dose- and time-dependent increase in p53 expression was detected, beginning after 12 hours' treatment at both IC50 and IC80. The maximum level of JNK expression (9-fold increase) was reached after 72 h at IC80. At this concentration the level of p53 increased 3-fold after 24 h and remained constant until the end of incubation. It is well established that the tumor-suppressor protein p53 controls cell growth through cell-cycle arrest and the induction of apoptosis [24]. It regulates different genes, such as p21 for the cell cycle and Bax for apoptosis [25]. This may well explain the cell cycle observed in response to MA treatment. Several authors have shown that the transcription of p53 brings about an accumulation of apoptotic cells in the G1 cell-cycle phase [26].

### Maslinic acid activates Bid and Bax and inhibits Bcl-2

JNK and p53 stimulate the mitochondrial apoptotic pathway, thus enabling direct protein-interaction-based activation or inhibition of the Bcl-2 protein family. JNK and p53 can also induce the pro-apoptotic Bcl-2 proteins by transcription or inhibit the transcription of anti-apoptotic Bcl-2 proteins. We examined the effect of MA on the expression of the Bcl-2 protein group. The proteins studied were the Bcl-2 anti-apoptotic protein Bcl-2 (48, 72 h), the Bcl-2 pro-apoptotic protein Bax (48, 72 h), and the Bcl-2 pro-apoptotic BH3 single-domain protein Bid (12, 24, 48 and 72 h).

Two different active fragments of Bid have been described: truncated Bid, or t-Bid, [27] and j-Bid [3]. Because these fragments are very small they remained undetected in the western blotting of this protein. Nevertheless, a decrease in the Bid-complete levels was detected in response to MA (Figure 2C). After 24 h this decrease was evident at both IC50 (32%) and IC80 (51%). The minimum level of Bid was reached after 48 h (56% at IC50 and 84% at IC80). These decreases remained until the end of treatment (48% at IC50 and 60% at IC80).

A cleaved form of Bid translocates to the outer mitochondrial membrane and promotes the oligomerization of Bax. Bcl-2 inhibition has also been reported in response to the mitochondrial translocation of cleaved Bid [27]. As the maximum levels of response to MA with regard to cell-cycle arrest and the stimulation of apoptosis were observed after 72 h we decided to analyse the expression of Bax and Bcl-2 after 24, 48 and 72 hours' treatment at both concentrations.

**Figure 3:**
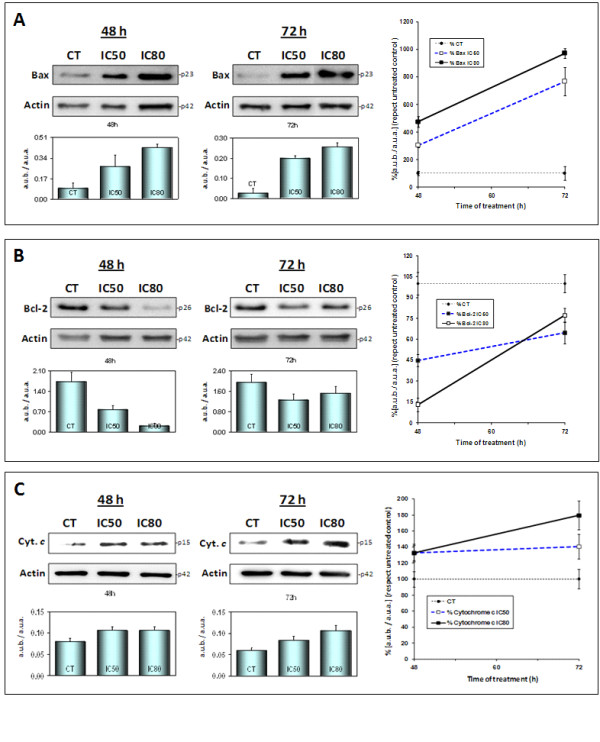
**Western blottings of the levels of Bax (panel A), Bcl-2 (panel B) and cytochrome-c (panel C) proteins**. HT29 cells were treated with maslinic acid at concentrations of IC_50 _and IC_80 _for 48 and 72 h. The levels of protein expression are expressed as arbitrary intensity units of each band compared to arbitrary intensity units of actin. The variations in relative percentages of the expression of Bax, Bcl-2 and cytochrome-c for each time and concentration are also shown. The values represent means ± SD of at least three separate experiments.

The results showed a dose-dependent increase in Bax expression over time (Figure 3A) and a concomitant decrease in the expression of Bcl-2 (Figure 3B). The maximum levels of Bax were reached at the end of incubation (6.5-fold at IC50 and 8.5-fold at IC80) (Figure 3B). As for Bcl-2, there was a marked decrease from 48 h to minimums of 55% at IC50 and 87% at IC80. These levels later increased somewhat but always remained lower than in the control cells until the end of the assay at 72 h (35% at IC50 and 23% at IC80). Both Bax activation and Bcl-2 inhibition are required for the release of mitochondrial apoptotic factors and the activation of the intrinsic apoptotic route.

### Maslinic acid induces the release of cytochrome-c and the activation of caspases

The activation of pro-apoptotic Bcl-2 proteins and inhibition of the anti-apoptotic Bcl-2 protein facilitate the release of cytochrome-c and other apoptogenic proteins from the intermembrane space of the mitochondria into the cytosol. Cytochrome-c, released in the presence of (d)ATP, binds to and activates the adaptor protein Apaf-1, which in turn recruits caspase-9, leading to the formation of apoptosome, the activation of caspase-9 and subsequent activation of executioner caspases -3 and -7 [28].

Here we have studied this effect after longer incubation times and at higher MA concentrations to find out more about its prolonged effect. Cytochrome-c levels were measured after 48 and 72 h at IC50 and IC80. The results showed that the release of cytochrome-c was both time- and dose-dependent, maximum levels being reached after 72 h at IC80 (Figure 3C).

**Figure 4:**
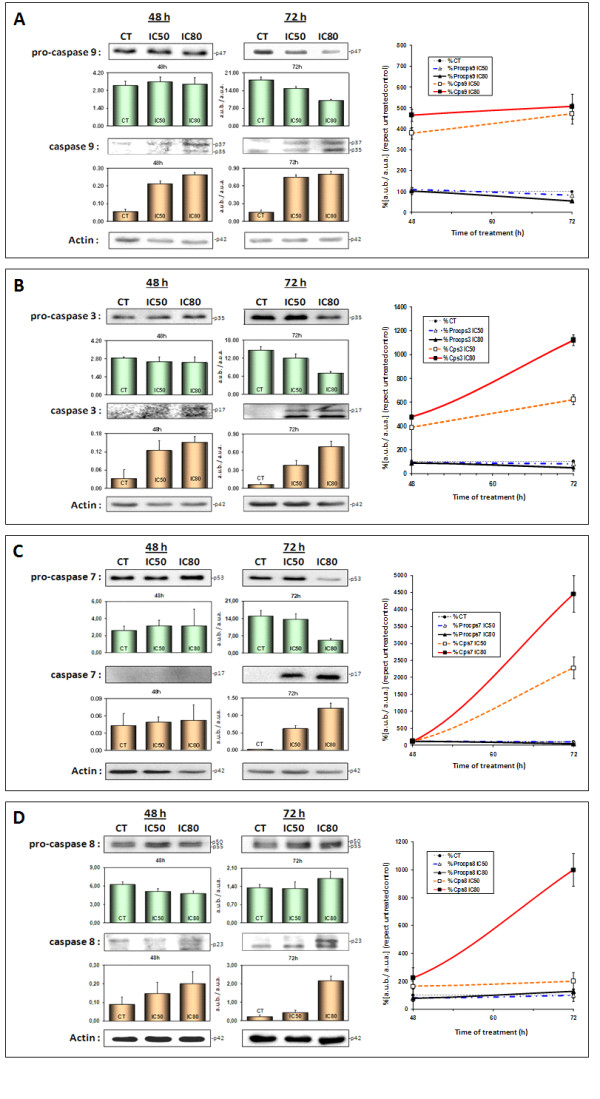
**Western blottings of the levels of pro- and caspase-9 (panel A), pro- and caspase-3 (panel B), pro- and caspase-7 (panel C), and pro- and caspase-8 (panel D) proteins**. HT29 cells were treated with maslinic acid at concentrations of IC_50 _and IC_80 _for 48 and 72 h. The levels of protein expression are expressed as arbitrary intensity units of each band compared to arbitrary intensity units of actin. The variations in relative percentages of the expression of proteins for each time and concentration are also represented in this figure. The values represent means ± SD of at least three independent experiments.

With regard to caspase activation, we looked at the expression levels of executor caspases -3 and -7, and initiator caspases -8 and -9. After 48 h caspase-9 was completely activated (3-fold at IC50 and 4-fold at IC80) (Figure 4A). This activation reached maximum activity after 72 h. The maximum levels of caspase-3 were reached after 72 h (5-fold at IC50 and 10-fold at IC80) (Figures 4B). Coinciding with maximum caspase-3 activation, we observed a significant activation of caspase-7 (20-fold at IC50 and 40-fold at IC80) (Figures 4C).

Finally, we observed a belated increase in the expression of caspase-8. The late activation of this protease has been described in several mitochondrial-mediated apoptotic mechanisms [29]. Our results showed clear activation of caspase-8 (8-fold) at the end of treatment at IC80 (Figure 4D). This belated caspase-8 activation may serve to amplify the initial mitochondrial apoptotic signaling via the JNK pathway. All these changes in caspase signaling explain the apoptotic phenomenon observed in the HT29 cell line in response to maslinic acid.

## Discussion

Apoptosis is one of the body's most potent defences against cancer; the pathogenesis of many forms of this degenerative disease is closely connected with aberrantly regulated apoptotic cell death. Different mechanisms that regulate apoptosis associated with mediators that trigger or inhibit cell death have led to the development of therapeutic strategies against cancer. It is now known that various triterpenes are able to intervene in such processes as DNA repair, cell proliferation, cell differentiation, angiogenesis and apoptosis [18,20,30]. Whilst in previous studies we have demonstrated clearly that maslinic acid did not affect the apoptosis of normal cells [18] we are able to propose here a plausible molecular mechanism by which this triterpene might induce its anti-tumoral and pro-apoptotic effects upon HT29 colon-cancer cells.

We have shown that a sequential pathway involving JNK, p53, the Bcl-2 protein family and caspase is activated in synchrony during the time in which MA mediates apoptosis. The pro-apoptotic JNK cascade induces apoptosis via the mitochondria-dependent pathway. The JNK-induced activation of the mitochondrial apoptosis pathway is not a secondary consequence of caspase activation, which proves the hypothesis that JNK is involved in the activation of the intrinsic mitochondrial apoptotic pathway [31]. We found a marked and significant increase in JNK expression (2.5-fold at 12 h) from the beginning of treatment with MA. The main result supporting our hypothesis is the induction of JNK before caspase-8 is activated and apoptosis occurs. This early induction of JNK may explain the activation of p53 and mitochondrial apoptosis mediated by the Bcl-2 protein family triggered by maslinic acid.

The function of p53 is also regulated by JNK phosphorylation [11]. In response to MA treatment, p53 was induced in a time- and dose-dependent way, reaching its maximum level after 72 h (9-fold increase). Several authors have reported that p53 controls cell-cycle arrest and apoptosis induction by regulating the expression of different proteins such as p21, Bax and Bcl-2 [32,33]. Other authors have described p53 expression in response to genotoxicity [34,35]. For these reasons we are confident in our assertion that the effects of MA upon HT29 cells are mediated in part by the expression of p53.

The over-expression of p53 in HT29 cells could well explain cell-cycle arrest, cell differentiation and the late induction of genotoxicity. Our results show that MA can induce apoptosis and cell-cycle arrest in this cancer cell line in a dose-dependent way. We found that the cell-cycle was arrested by 22% with IC80 and 18% with IC50. Genotoxicity was not significant until after 48 hours' treatment (6-fold at IC50). This delay was related to the behaviour of p53 activation. An analysis of the cell cycle also revealed that treatment with MA brought about the formation of apoptotic bodies. The apparition of a hypodiploid peak is clear after 96 h at IC80. Furthermore, the FS/SS graph shows the appearance of cell fragments. Our results demonstrate that the apoptotic effect is mediated by the activation of the mitochondrial pathway.

It has also been shown that p53 itself translocates to mitochondria, where it activates the mitochondrial apoptotic pathway by binding to the anti-apoptotic proteins Bcl-2 and Bcl-xL [33]. An accumulation of p53 in the cytosol can act in a similar way to BH3-only proteins, thus inducing the oligomerization of Bax [32]. Finally, p53 leads to the expression of pro-apoptotic proteins, including members of the BH3-only group of Bcl2-related proteins such as Bid, which can trigger the mitochondrial apoptotic pathway [36]. Anti-apoptotic proteins such as Bcl-2 have been reported to be phosphorylated and inactivated by JNK. In addition, it has also been reported that the activation of JNK-mediated Bid produces the active fragment j-Bid [3], whereas pro-apoptotic Bax is not activated in JNK-deficient cells [6]. We have also identified the Bcl-2 proteins involved in maslinic-acid-induced apoptosis.

Members of the three subfamilies of Bcl-2 proteins are involved in the stimulation of apoptosis by MA. We observed a decrease in the time- and dose-dependence of Bid-complete levels, reaching 84% at IC80 after 48 h. Bax activation also reached its maximum after 72 h (8.5-fold at IC80). Finally, Bcl-2 inhibition was obvious after 48 hours' treatment (87% at IC80). These data show that mitochondrial apoptosis is activated by MA, probably in response to the induction of JNK and p53, all of which leads to the levels of mitochondrial cytochrome-c remaining high until 72 hours' treatment at both IC50 and IC80.

Other triterpenoids such as ursolic acid, CDDO and betulinic acid have been reported to stimulate similar apoptotic mechanisms. Ursolic acid induces apoptosis via the mitochondrial intrinsic pathway with alterations of the Bax/Bcl-2 balance in M4Beu cells [37] and its anti-carcinogenic properties have also been described [38]. Another triterpene, CDDO, which triggers the cleavage of Bid and the release of mitochondrial cytochrome-c, is blocked by the over-expression of Bcl-xL or Bcl-2 [22,39]. It has also been reported that CDDO stimulates JNK, the activation of caspase-8, loss of mitochondrial transmembrane potential, release of cytochrome-c and cleavage of caspase-3 [20]. Finally, CDDO up-regulates p21 and down-regulates cyclin D1 and Bcl-2, thus inducing cell-cycle arrest and apoptosis in MDA-MB-435 tumor cells [40]. CDDO-Me rapidly activates the JNK apoptosis pathway, whereas the action of a JNK-specific inhibitor blocks CDDO-Me induced apoptosis [21]. This synthetic triterpenoid does not alter the expression levels of Bcl-2 and Bcl-xL but does raise Bax expression levels [41], leading to the suppression of the expression of NF-kB-regulated gene products [42].

The induction of apoptosis by betulinic acid involves several mitochondrial perturbations, such as the release of cytochrome-c and activation of caspase-8 [43]. Furthermore, it has been reported that the protein Smac is released during the induction of apoptosis by betulinic acid, whereas with other tumoral cells it provokes the down-expression of Bcl-2, thus blocking the release of anti-apoptogenic molecules [44]. In addition, the formation of reactive oxygen species that modulate Bcl-2 and Bax levels during the action of betulinic acid has also been detected [45].

Other terpenoids, such as amooranin for example, induce apoptosis in MDA-468 cells via the activation of caspases -9, -3, and -8, the cleavage of Bid and the release of cytochrome-c from the mitochondria, concomitant with the up-regulation of p53 and Bax and down-regulation of Bcl-2 [46]. In addition, an increase in the Bax/Bcl-2 ratio and a decrease in mitochondrial membrane potential have been reported as being involved in the induction of apoptosis for alisol B acetate [47].

## Conclusions

Our own results show that treating HT29 adenocarcinoma cells with MA causes cascade signaling in the induction of caspases, which in turn governs the mechanisms for inducing apoptosis. Initiator caspase-9 and executor caspase-3 were activated significantly after 48 h, reaching their maximum levels after 72 h., The initiator caspase-8 and executor caspase-7, on the other hand, were not activated until 72 h. This belated activation of caspase-8 is almost certainly related to the strengthening and consequent amplification of the initial mitochondrial apoptotic signaling.

**Figure 5:**
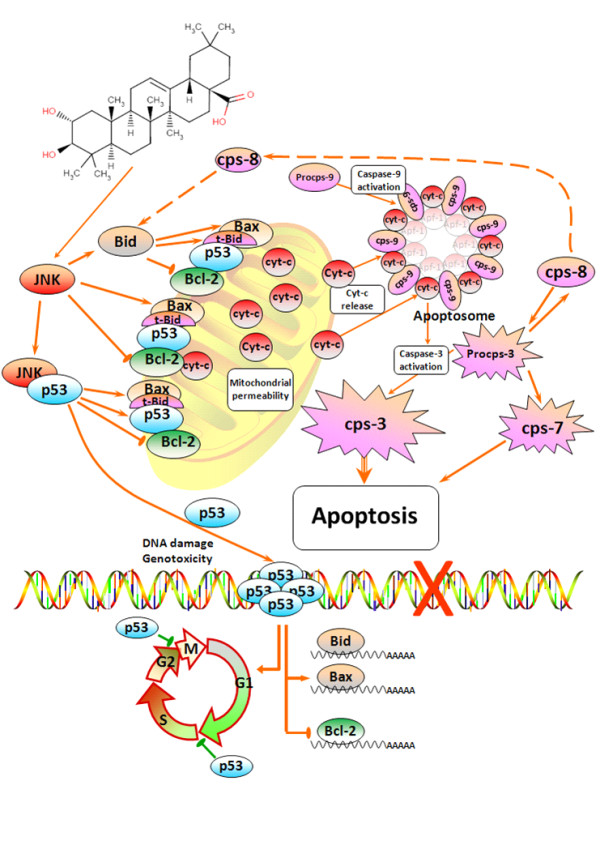
**Schematic representation of the plausible molecular mechanism proposed for the induction of apoptosis by maslinic acid in HT29 colon-cancer cells**. This molecular mechanism is regulated via the induction of JNK and p53, resulting in mitochondrial disruption, the release of cytochrome-c and finally the activation of a cascade of caspases.

According to these results we propose the following mechanism for the apoptotic effect of MA upon HT29 colon-cancer cells (Figure 5). Initially MA induces both JNK and p53, thus provoking cell-cycle arrest and at the same time a delay in the induction of genotoxicity. Subsequently, in response to this activation of JNK and p53, the expression of the pro-apoptotic Bcl-2 proteins Bax and Bid is enhanced whilst that of Bcl-2 is inhibited. The apoptotic mitochondrial pathway is thus triggered, producing mitochondrial disruption and the activation of caspase-9, which finally leads to the activation of caspases -3, -8 and -7. Further studies inhibiting these routes in different colon-cancer cell types will be needed to confirm the proposed mechanism and justify its general validity.

The apoptotic potency of MA suggests that it may be an effective compound in therapy for the treatment of colon cancer. Within this context, our most recent results with APC-min mice, animals with a positive cancer-colon phenotype (data not shown), have shown a considerable decrease in the number, development and extension of tumors in the animals treated with maslinic acid.

## List of abbreviations

CDDO: 2-cyano-3,12-dioxoolean-1,9-dien-28-oic acid; DAPI: 4',6-diamidino-2-phenylindole; DMEM: Dulbecco's modified Eagle's medium; DMSO: dimethyl sulfoxide; FACS: fluorescence-activated cell sorter; FCS: fetal calf serum; MA: maslinic acid; PI: propidium iodide.

## Competing interests

The authors declare that they have no competing interests.

## Authors' contributions

FJR-Z carried out in all assays relating to the study and participated in the drafting of the manuscript. GP-P, DL and EER-P participated evenly in all assays relating to the study. MC and JAL conceived of the study, were responsible for the design and coordination of this study and drafted the manuscript. All authors read and approved the final manuscript

## Pre-publication history

The pre-publication history for this paper can be accessed here:

http://www.biomedcentral.com/1471-2407/11/154/prepub
